# Talent Development Environments in Football: Comparing the Top-Five and Bottom-Five-Ranked Football Academies in Norway

**DOI:** 10.3390/ijerph18031321

**Published:** 2021-02-01

**Authors:** Kristian Gangsø, Nils Petter Aspvik, Ingar Mehus, Rune Høigaard, Stig Arve Sæther

**Affiliations:** 1 Department of Sociology and Political Science, Norwegian University of Science and Technology (NTNU), 7491 Trondheim, Norway; Kristian.gangso@tffk.no (K.G.); nils.petter.aspvik@ntnu.no (N.P.A.); ingar.mehus@ntnu.no (I.M.); rune.hoigaard@uia.no (R.H.); 2 Department of Sport Science and Physical Education, University of Agder, 4604 Kristiansand, Norway

**Keywords:** talent development environment questionnaire, football, youth sports

## Abstract

Background: The aim of this study was to examine junior-elite football players’ perception of their talent development environment by comparing clubs ranked as the top-five and bottom-five in the 2017 Norwegian academy classification. Methods: In total, 92 male junior-elite football players recruited from under-19 teams from five professional football club academies took part in the study. The Talent Development Environment Questionnaire (TDEQ-5; Martindale et al. 2010) was used to measure the players’ perceptions of their team environment. Results: The subscale long-term development focus and support network had the highest score and indicated that they perceived that the environment was high quality with respect to those factors. Players from the top-five-ranked clubs perceived their development environments to be significantly more positive with respect to holistic quality preparation, alignment of expectations, communication and, compared to players from the bottom-five-ranked clubs. Conclusions: The players’ perceptions of the talent development environment seem to be in alignment of the academy classification undertaken by the Norwegian top football association.

## 1. Introduction

The identification and development of talented individuals in football has become increasingly important over recent years, as the standards of sporting performance have grown [1]. Subsequently, the cumulative body of research examining talent identification (TID) [2], and the initiation or adoption/implementation of talent development (TD) programs to achieve sporting excellence has significantly increased [3,4,5]. Despite this increased interest, some of the newest reviews on TID/TD have stated that the field still “lacking robust research evidence” [6] (p. 10). Similarly, Johnston et al. [7] summarized the research on longitudinal and retrospective studies between 1990 and 2015 that examined differences in performance variables between highly skilled and less-skilled athletes, by stating “Overwhelmingly, findings from this review revealed inconsistent and unreliable predictors and demonstrated a fairly homogenous body of research on TID in elite-level sport” (p. 107). Others have argued that this research has demonstrated that spending time on identifying sport talents may not be productive [8]. 

Irrespective of TID criteria, most professional football clubs want to provide high-quality talent development environments and strategies arranged in academies to provide players for a club’s professional team [9]. One of the first to introduce the importance of talent development environment (TDE) was Gagné [10,11] who highlighted the three environmental considerations: milieu, individuals and provisions as well as the interplay between a whole host of different factors such as natural abilities, intrapersonal characteristics, environmental features, learning opportunities and chance. Martindale et al. [12] furthermore highlighted in their review of the literature five key generic features to facilitate a talent development environment: long-term aims and methods, wide-ranging coherent messages and support, emphasis on appropriate development rather than early selection, individualized and ongoing development, and finally integrated, holistic, and systematic development. This means that in addition to focusing merely on intrapersonal factors (e.g., physical traits, skills, attitude), TDE factors should be identified and enhanced to effectively nurture talented athletes. Martindale et al. [13] interviewed coaches of world champions and Olympians and found the same factors as vital in the talent development process. With the use of a holistic ecological approach grounded in Bronfenbrenner’s theory [14], system theory work [15], and organizational psychology [16], Henriksen et al. [17] developed a talent development environments (ATDE) and addition an environment success factors (ESF) model. The component in ATDE model is structured into two levels (micro- and macro-) and two domains (athletic and non-athletic), complemented by the past, present and future of the ATDE. Using a qualitative approach, they found that a high-talented environment was characterized by a high degree of cohesion, with the relationship between current and prospective elite athletes at its core. Furthermore, they reported that a strong organizational culture, characterized by values of open co-operation, individual responsibility and a focus on performance process was factors that compensated for lack of resources. Several studies have used the ATDE and ESF models in soccer, and have found that common features among successful environments (production of players to professional level) was player accountability for development, video feedback to develop tactical skills and a common playing-style philosophy [18,19,20,21]. Further, the findings from these studies suggest that, in successful environments, different domains seem to work together and equip players with the resources needed both on the pitch and in life in general (social networks, dual careers). Another research avenue trying to capture the essence of characteristics of TDE has been conducted by Martindale and colleagues [22,23]. They have developed the talent development environment questionnaire (TDEQ), which measures key holistic and generic processes involved in the effective development of “talented” athletes. The questionnaire contains five dimensions: long-term development (e.g., the extent to which developmental programmes are specifically designed to facilitate athletes´ long term success), holistic quality preparation (e.g., the extent to which intervention programmes are prepared both inside and outside of sports settings), support network (e.g., the extent to which a coherent, approachable, and wide-ranging support network is available for the athlete in all areas), communication (e.g., the extent to which the coach communicates effectively with the athlete in both formal and informal settings) and alignment of expectations (e.g., the extent to which goals for sport development are coherently set and aligned) (see Martindale et al. [22,23] and Li et al., [24] for a more detailed description of the five dimensions). TDEQ has been widely used in different sport context (e.g., golf, soccer, sailing) and countries (e.g., Singapore England, Spain, China) and seems to be a valid and sound measure investigating talent environment [5,25,26,27,28,29,30].

From a more applied organizational perspective, the TDE has been highlighted by the national football federation which has made them introduce academy classifications in order to measure, rank and assure the quality of the clubs’ academy environments [31]. In Norway the introduction of an academy classification is new. The first football academy classification was undertaken in 2017, consisting of ten factors (see methods for descriptions). Based on the ranking of these ten factors, each academy is given stars from one to five, indicating the overall quality of the academy. Since these types of academy classifications are given increased value by football federations, relating them to the perceived TDE in the academies could give insight into their impact on development. Even though the TDEQ has been used in different countries and contexts, few of these studies have been done in the elite level context, and none comparing environment on an elite level. The aim of this study was to investigate Norwegian junior-elite football players´ perception of their talent development environment with the use of the TDEQ-5 questionnaire. The second aim was to compare environmental differences between academies ranked as top-five and bottom-five according to the academy classification.

## 2. Materials and Methods

### 2.1. Procedure and Participants

A sample of 92 male elite youth football players between 17 and 19 years old (mean age 17.02, SD 0.94) enrolled in a football academy from five professional Norwegian clubs participated. Three clubs were ranked as top-five clubs and two clubs were ranked bottom-five clubs. The purpose of the study was described for the whole team, and each player were provided with a letter of information and a consent form to be signed. Participants were informed they could withdraw from the study at any given point. The players completed the questionnaire after a training session with the first author present. The researcher encouraged participants to respond to the questionnaire honestly. It took participants approximately 12 min to complete the survey. Ethical clearance was in accordance with and approved by the Norwegian Social Sciences Data Services. 

#### The Context

In Norway, the first football academy classification was undertaken in 2017, consisting of ten factors, (1) board anchoring, management, and employees, (2) player logistics, (3) planning, (4) competence, (5) training process, (6) match platform, (7) school/football, (8) collaboration models, (9) productivity and (10) economy and facilities [32]. The academy classification gives an overall evaluation of the academy´s standards both related to sporting and non-sporting aspects. Based on the ranking of these ten factors each academy could potentially reach 165 points, which indicates that this is a five stars to the academy, while forty points indicates a one-star academy. In order to be ranked as a top-five academy in 2017, there was a 123.5-point limit, with the highest-ranked academy given 129.5 points. The bottom-five academies scored between 61.2 and 87 points. The classification did not include any psychometric properties.

### 2.2. Measures

#### Talent Development Environments

A Norwegian version of the TDEQ-5 [22] was used to measure participants´ perceptions of the five talent development environment dimensions. The questions were forward and backward translated by two of the native speaking Norwegian authors who were fluent in English. The scale has the following five subscales: support network (four items), an item example: “I can pop over to see my coach or other support staff whenever I need to”; long-term development focus (five items), an item example: “My training is specifically designed to help me develop effectively in the long term”; holistic quality preparations (seven items), an item example: “My coach rarely talks to me about my well-being” communication (four items), an item example: “My coach and I often try to identify what my next big test will be before it happens” and alignment of expectations (five items), an item example: “I regularly set goals with my coach that are specific to my development”. All items were presented on a 6-point Likert scale ranging from 1 (strongly disagree) to 6 (strongly agree). Negatively worded items are reverse scored. 

### 2.3. Data Analysis

Internal consistency for each dimension was examined using Cronbach´s alpha (α) coefficients, where anything above 0.60 is considered adequate and above 0.70 good [33]. Means and standard deviations were calculated for the dimensions of the TDEQ-5. In addition to correlation analysis Student t-tests were performed for all main variables examining any potential differences between academies ranked as top-five (N = 52) and bottom-five (N = 40) in the academy classification of 2017. The significance level was set to *p* < 0.05. 

## 3. Results

All TDEQ dimensions had adequate alpha values; Support network (α = 0.69). Long-term development focus (α = 0.68), Holistic quality preparation (α = 0.82), Communication (α = 0.81) and Alignment of expectations (α = 0.75), even though the dimensions Support network and Long-term development focus was slightly below appropriate level, but still in line with earlier TDEQ studies.

The results showed that the players rated the five development environment features between 3.94 and 4.66, with the highest rating on support network 4.66 (0.85), and long-term development focus 4.63 (0.66) dimensions (see Table 1). The correlations between the dimensions were all significant, with correlations between r = 0.44 and 0.72. In comparing the top-five and bottom-five-ranked clubs, significant differences were found for the following three dimensions: holistic quality preparation, communication, and alignment of expectations.

## 4. Discussion

The aim of this study was to investigate Norwegian junior-elite football players´ perception of their talent development environment and furthermore to compare environmental differences between academies ranked as top-five and bottom-five according to the academy classification.

The subscale long-term development was the highest rated as in earlier TDEQ studies on TDE´s [5,25,26,27,28,29,30]. Compared to earlier studies the academy players in this study rated support network, considerably higher compared to other studies on non-professional male athletes [5,25,26,27,28,29,30]. This result is however similar to a study of female professional football players [34]. These results could be related to this study´s sample of professional club academies, expected to offer and invest in highly skilled development and performance environments [1]. Another explanation could be contextual. This study was done within a Norwegian sports context which could be considered different compared to other European countries in term of focus in their talent development environments. The Norwegian sporting model connecting both mass and elite sport in a ‘sport for all’ concept and the sport is based on a social democratic approach focusing on equality [35]. Furthermore, in Norwegian sport the importance of social support from both coaches and parents is highly valued, and introduced in youth sport, and continually focused on when the athletes are introduced to more professional environments [36].

Even so, the significant differences between the high- and low-ranked academies on the subscales: holistic quality preparation, alignment of expectations, and communication, indicate differences in the club’s ability to provide stimulating and development centered environments. The higher score for holistic quality preparation among the players in the highest-ranked academies indicates that the academies are better organised in relation to preparation and life balance. For example, a close collaboration between school and academy (making a dual career) has been found to be challenging, but crucial for optimal development [19]. Another explanation could be that the high ranked academies are well known for their ranking and thereby are both expected and perceived to give such a holistic approach [21]. Even so, since the academy classification is so broad, covering a total of ten factors both related to sporting and non-sporting aspects, it would also be natural to assume that an academy which is well adjusted to a holistic approach on one factor, is also systematic and holistic in others. The importance of a holistic approach is also vital related to the approach of the coaches in the academies, described as one of the most important actors in the talent development process [36]. Coaches have been related to issues such as the perception of need-support [37], social support when facing stressors [38] and burnout [39].

The subscale alignment of expectations and communication dimensions indicated that coaches in high-ranked academies are more able to include the players in their own developmental process, such as training, planning and goal setting and, in addition, communicate the demands and expectations necessary to become an elite football player. This is in line with earlier studies which have found this to be an essential factor for the players’ ability to take responsibility for their own development [18,20,21], stimulating self-regulated learning and subsequently increasing their performance.

Even if the results partly support the academy classification, we need to keep in mind that the classification is an overall evaluation of the academy. This can be a challenge since it covers sporting and non-sporting aspects, and as such gives a somewhat skewed picture of the proximal and direct sport quality of the academy, because the total score of the classification could rely primarily on more ‘distal’ factors (e.g., total economy, organizational aspect, recruitment strategies).

When investigating talent development environments, we need both quantitative and qualitative approaches and our opinion is, therefore, that a combination of TDEQ and ATDE is desirable. Studies utilising Henriksen et al.’s [17] ATDE and ESF models have highlighted that successful ATDEs also share a number of features compared to the TDEQ-5. The ESF success factors were long-term development rather than short-term success (TDEQ long-term development focus), support for sporting goals by the wider environment (TDEQ alignment of expectations/support network), opportunities for inclusion in a supportive training community/role models (TDEQ communication), and the integration of efforts between sport, school, family and other components of the environment (TDEQ holistic quality preparation) [18,19,20,21]. Both approaches cover different aspects, but also identify similar success factors, giving a broader perspective and more nuanced knowledge about talent development environments.

### Limitations and Implications

There are some limitations we need to keep in mind before generalizing the results of this study. Firstly, the cross-sectional design gives a snapshoot, and investigating causality is not applicable. Therefore, a longitudinal research design using different methods to investigate long-term effects of different talent development environments are warranted. Secondly, more research using TDEQ-5 is necessary; the questionnaire should be further validated and psychometrically tested in different sports. We recommend that future research should combine the approaches from Henriksen et al. [17] and Martindale et al. [22] in an attempt to get deeper insight into TDEs and investigate TDE in relationship outcome variables such as: stress, performance, coach behavior, motivation and health related variables.

## 5. Conclusions

The findings show that the two dimensions, long-term development focus and support network were the highest ranked dimensions in the academies. Players from the top-five-ranked clubs perceived their development environments to be significantly higher on holistic quality preparation, alignment of expectations and communication, compared to players from bottom-five-ranked clubs. The players’ perceptions of the talent development environment seem to be in alignment with the academy classification undertaken by the Norwegian top football association. This study indicates that the bottom-five academies should increase their focus on the holistic quality preparation, alignment of expectations and communication dimensions to improve their TDE.

## Figures and Tables

**Table 1:** Mean Value for the Five Dimensions in TDEQ-5 and Group Differences Among the Five Dimensions Comparing the Top-Five and Bottom-Five Academies.

Variables	High		Low		
	M	SD	M	SD	t
Support network	4.73	0.84	4.53	0.88	−1.08
Long-term development focus	4.74	0.71	4.50	0.56	−1.81
Holistic quality preparation	4.44	0.93	3.91	0.90	−2.72 **
Communication	4.17	0.81	3.78	1.03	−2.03 *
Alignment of expectations	4.17	0.82	3.64	0.85	−3.00 **

* *p* < 0.05 ** *p* < 0.01, low-ranked academies different from high-ranked academies.

## Data Availability

The data are not publicly available as data sharing was not included within the original study ethics submission or participant consent form.
